# Recent Issues in Varicella-Zoster Virus Latency

**DOI:** 10.3390/v13102018

**Published:** 2021-10-07

**Authors:** Peter G. E. Kennedy, Trine H. Mogensen, Randall J. Cohrs

**Affiliations:** 1 Institute of Neuroscience and Psychology, University of Glasgow, Glasgow G61 1QH, UK; 2 Department of Infectious Diseases, Aarhus University Hospital, 8000 Aarhus, Denmark; trine.mogensen@biomed.au.dk; 3 Department of Biomedicine, Aarhus University, 8000 Aarhus, Denmark; 4 Department of Neurology, University of Colorado School of Medicine, 80045 Aurora, CO, USA

**Keywords:** varicella-zoster, virus, latency, reactivation, epigenetics, gene expression, immunity, neuron

## Abstract

Varicella-zoster virus (VZV) is a human herpes virus which causes varicella (chicken pox) as a primary infection, and, following a variable period of latency in neurons in the peripheral ganglia, may reactivate to cause herpes zoster (shingles) as well as a variety of neurological syndromes. In this overview we consider some recent issues in alphaherpesvirus latency with special focus on VZV ganglionic latency. A key question is the nature and extent of viral gene transcription during viral latency. While it is known that this is highly restricted, it is only recently that the very high degree of that restriction has been clarified, with both VZV gene 63-encoded transcripts and discovery of a novel VZV transcript (VLT) that maps antisense to the viral transactivator gene 61. It has also emerged in recent years that there is significant epigenetic regulation of VZV gene transcription, and the mechanisms underlying this are complex and being unraveled. The last few years has also seen an increased interest in the immunological aspects of VZV latency and reactivation, in particular from the perspective of inborn errors of host immunity that predispose to different VZV reactivation syndromes.

## 1. Introduction

Varicella-zoster virus (VZV) is a pathogenic human alphaherpesvirus which is a significant cause of morbidity. VZV causes a primary infection, usually in children, called varicella (chicken pox) following which it establishes ganglionic latency in neurons [1,2]. Latency is established in ganglia throughout the entire neuroaxis including the dorsal root ganglia (DRG), trigeminal ganglia (TG) and also autonomic ganglia including the enteric ganglia [2,3]. After a variable period, which can span several decades, VZV may reactivate from the latent state in human ganglia to cause the well-recognised syndrome of herpes zoster (shingles) which is an extremely painful vesicular rash with an anatomical distribution of a particular sensory dermatome [3]. While viral reactivation may occur spontaneously, it can also follow one or more triggering factors such as diminished cell-mediated immunity to the virus as occurs with older age or immunosuppression due to drug treatment or disease, X-Ray irradiation, infection, trauma or malignancy [2].

Post-herpetic neuralgia (PHN), a condition highly refractory to treatment, is the most well-known complication of herpes zoster, causing severe and constant pain in the affected dermatome lasting longer than three months after the zoster rash [3]. It has become increasingly recognised that the disease spectrum caused by VZV reactivation is much wider than previously thought [2,4]. As well as causing PHN, VZV reactivation may also lead to a wide variety of neurological conditions including a VZV vasculopathy, myelitis and focal motor weakness, a syndrome called *zoster sine herpete*, which is the presence of herpes zoster in the absence of a rash, Ramsay Hunt syndrome and meningoencephalitis, [4,5]. A possible diagnosis of VZV reactivation-induced neurological disease should now be considered in all cases of undiagnosed acute, subacute or chronic brain or spinal cord syndromes, particularly if there is an accompanying cerebrospinal fluid (CSF) pleocytosis [6]. 

The structure of VZV is now well established. It is, in common with the other herpesviruses, a double-stranded DNA virus and it has a genome comprising approximately 125,000 base pairs. Though it has long been thought that its genome contains 68 open reading frames (ORFs), with three of them repeated, it is possible that there may be, in reality, more than this number of ORFs, with a recent genome-wide transcriptomal study suggesting that VZV may encode more than just 68 ORFS [7]. Furthermore, a transcript presence and the corresponding ORF coding potential are not synonymous [8]. A particular biological feature of VZV is that it is highly cell associated in vitro, making the generation of cell-free virus difficult to grow in high titer, and it also only replicates in human cells [2]. It has not been possible to produce good animal models of VZV latency, in particular because of the human specificity of VZV, and this has hindered attempts to understand the VZV latency process in vivo. However, because of the limitations of studying viral latency in post-mortem tissues (see below), future studies might rely on induced pluripotent stem cells and/or neuronal or brain organoids [9]

In this overview, the intention is to highlight some recent developments in our thinking about alphaherpesvirus latency, and VZV in particular; thus, this review will be selective rather than comprehensive. Different, but related, aspects of VZV latency will be considered, namely viral gene expression in latently infected ganglia, epigenetic control of viral transcription and immunological aspects of VZV latency and reactivation, the latter mainly based on studies of patients with severe VZV infection and underlying inborn errors of immunity.

## 2. The Nature of VZV Latency

As indicated, after primary infection, VZV readily establishes latency in neurons [1,10,11] of the peripheral nervous system [12,13] from which the virus can reactivate to produce progeny virions along with cutaneous and neurological disease, especially in the elderly or immunocompromised [2,14,15]. Thus, the virus can persist in isolated populations (the Tiriyo, an isolated Amazon tribe) without the presence of overt disease [16]. This clinical definition of latency has been updated with each major advance in techniques detecting evidence of the virus [17]. Thus, latency is now characterized by the maintenance of the virus genome in an endless (episomal) configuration, limited transcription of virus genes and the ability of the virus to reactivate resulting in the production of progeny virions [18].

There are two main reasons why knowledge of VZV gene expression during ganglionic latency is potentially important. First, since the functional properties of several VZV genes are known, it is possible that identification of a specific viral transcript that is expressed during latency may give clues as to its functional importance in the process. Second, if it is known that a particular VZV transcript (or transcripts) is or are present during latency, then that may represent a potential therapeutic target in individuals with recurrent VZV reactivations producing herpes zoster. However, it should be appreciated that if a lytic transcript is detected, then it may function completely differently in latency. Moreover, knowledge of a specific transcript being present would only be an initial stage in potential drug development, which would certainly not be guaranteed, and further knowledge including the identification of a corresponding protein would be important.

It is established that latent VZV, such as herpes simplex virus-1 (HSV-1), is present episomally in a circular form in ganglionic neurons [19]. While it is clear that viral gene expression in ganglionic neurons is restricted, the main issue of uncertainty and contention has been the actual extent of this viral transcript restriction. In this context, a key issue is that since such studies must of necessity be performed on human ganglia that have been removed at autopsy, the very process of death may itself predispose to viral reactivation. It follows logically from this that the extent of viral gene expression detected in latently infected ganglia may well be dependent on the interval between death of the individual and removal and analysis of the ganglionic tissue. Recent evidence suggests that this may indeed be the case. An early study [20] used a cDNA library from the mRNA of latently infected human ganglia to show that VZV gene transcription in these ganglia was limited to VZV genes 21, 29, 62 and 63. A subsequent study using in situ hybridization techniques [21] confirmed these findings, and then both laboratories combined their expertise to report the transcription and translation of VZV gene 66 in human post-mortem ganglia [22]. Overall, the results of such studies identified the presence of VZV gene 63-encoded transcripts as being the hallmark of VZV latency [19]. However, all of these studies, even though they used different techniques, were hampered by the delay of up to 24 hours between the death of the individual and the removal and analysis of the ganglia. Clarification of this difficult time delay issue and its effects on viral gene expression has now been provided over the last few years. Recently, Depledge and colleagues [23] reasoned that only autopsy tissues removed within a very short time after death would be likely to give a true picture of VZV gene expression during latency since VZV would already have reactivated after longer delays. Accordingly, they obtained post-mortem ganglia very early, within about 6 hours after death of the individuals, before any viral reactivation was likely to have taken place, and detected just two viral transcripts using a highly sensitive enriched RNA-Seq method. As well as detecting VZV ORF 63, as expected, they also detected a spliced latency associated VZV transcript (VLT) that mapped antisense to the viral transactivator gene 61 [23]. This was a major new finding in the VZV ganglionic latency field and is discussed further below in the epigenetics section. The VLT is expressed in human TG neurons and encodes a protein with late kinetics in productively infected cells in vitro and in shingles skin lesions [23]. These authors followed up this study by clarifying the relation of VZV ORF 63 expression to the expression of the VLT. They reported [24] that during VZV reactivation from the latent state, broad viral gene expression was induced by a VLT-ORF 63 fusion transcript. It is hoped and expected that over the next few years, further insights into our understanding of VZV gene expression during latency will be forthcoming.

## 3. Establishment of Alphaherpesvirus Lytic and Latent Infection

The result of lytic infection and the following virus reactivation is the same: the production of progeny virions. However, this result is obtained through pathways that are different at critical stages. The following discussion is centered on VZV but includes results obtained with another alphaherpesvirus (herpes simplex type 1; HSV-1). VZV and HSV-1 both establish latent infection in neurons of the peripheral nervous system [25], even within the same cell [26] from which both viruses can reactivate and even in response to the same stimuli [27]. Both viruses have similar genomic architecture consisting of unique long and short stretches of DNA bounded by segments of inverted repeated DNA [19]. Both viruses share a majority of genes in similar mapping locations [19]. However, VZV diverged from HSV-1 about 120 million years ago [28], and 30 million years ago the virus successfully deleted approximately 25 kilobases located in the invert repeat of the unique long segment [29]. Furthermore, the VLT encodes a protein unlike the HSV-1 LAT so it cannot be assumed that HSV-1 should be a paradigm for alphaherpesvirus latency and reactivation. Additionally, HSV-1 reactivations are multiple at short intervals as opposed to VZV reactivations which occur after decades [3]

Since VZV is strictly a human pathogen [30] and, in culture, is highly cell associated [31], investigation of the virus has been hindered. While the increased use of cultured human neurons generated from induced pluripotent or embryonic stem cells or from autopsy derived samples has provided a viable platform to study virus latency and reactivation [9,32,33,34,35,36,37,38], there is currently no technique to overcome the VZV infectious to defective particle ratio that approaches 1:20,000 [39], and synchronized infections are extremely difficult to attain. An in-depth analysis of the epigenetic state of the VZV genome during latency and reactivation is probably on the verge of an explosive increase. 

During lytic infection, HSV-1 capsids contact the host nuclear pore via the virus portal protein and the encased virus DNA under 30 atmospheres of internal pressure is explosively injected into the nucleoplasm [40,41]. At this time, the virus DNA is free of proteins. The success of site-directed mutagenesis, using bacterial artificial chromosome technology, has shown that protein-free HSV-1 and VZV DNA is infectious; however, the efficiency is increased when viral immediate-early proteins are present [42,43,44]. The incoming virus DNA rapidly associates with histones that ultimately contain posttranslational modifications (PTM) indicative of silenced genes [45]. These nucleosomes are not ordered but are highly unstable, mobile and situated throughout the virus genome [46,47]. Virus tegument proteins (VP16 for HSV-1 and IE62 for VZV) delivered into the cell during virus entry recruits host cell factor-1 (HCF-1) along with histone methyltransferases Set1 and MLL1 to the specific virus promoters where silencing histone modifications (lysine 3 on histone protein 3; H3K4) are removed and H3K4 trimethylations, a more transcriptionally permissive PTM, is added [48,49]. Thus, fully functional tegument proteins, introduced during initial virus infection, help to overcome an initial host mechanism designed to recognize and silence foreign (protein-free) DNA. As a result, the lytic program begins with transcription of immediate-early genes from cognate promoters made accessible to transcription factors by chromatin remodeling [50]. The immediate-early genes are principally involved in inactivating further host anti-DNA defenses and priming the cell for further steps in the virus lifecycle that ultimately results in assembly and release of progeny virions.

Based on these considerations, latent infection can also be seen as host defenses overcoming the virus lytic program resulting in silencing of the virus and halting the disease process. Alternatively, latency can be seen as the virus remaining silent until an immunologically naïve population arises, and further virus spread is possible. This teleological approach is based on the premise that the virus genome must remain transcriptionally silent (to avoid immune clearance) except for a region of the virus genome that can sense the status of the host neuron. However, it should be appreciated that there are many critical differences between initial lytic infection and reactivation, which could be related to latency 

## 4. Alphaherpesvirus Latency Mediated through Expression of Viral Latency Transcripts

During latency, both VZV and HSV-1 contain a region that remains transcriptionally active. The varicella latency transcript (VLT) for VZV and the latency associated transcript (LAT) for HSV-1 are transcribed during latency when the rest of the virus DNA contains histones with PTM indicative of heterochromatin and, thus, is transcriptionally silent. Both VLT and LAT are spliced transcripts mapping antisense to the immediate-early virus gene involved in disarming the host response to foreign DNA; open reading frame (ORF) 61 encoding immediate-early protein 61 (IE61) for VZV and RL2 encoding infected cell protein 0 (ICP0) for HSV-1. VZV VLT is a set of transcripts that originate from the same transcriptional start site (TSS) but differ in intron and transcription termination site usage [7]. Notably, within the set of VLT transcripts, ORF 63 is retained as an exon. The retention of ORF 63 explains earlier results that indicated that sole transcription of ORF 63 was the hallmark of latency. Since the earlier PCR-based studies were designed to identify only transcripts mapping to then known VZV genes, the VLT region was not included. While independent evidence from simian varicella virus, the primate counterpart to human VZV, suggested the presence of VLT [51], it was not until an unbiased transcriptome map was generated from RNA extracted from latently infected human trigeminal ganglia and enriched for virus mRNA that the previously detected ORF63 transcripts were determined to be a retained VLT exon [23]. HSV-1 LAT is also a set of transcripts that include an 8.3 × 10^3^ nucleotide primary transcript from which a 1.9 × 10^3^ nucleotide intron is removed and stabilized against rapid degradation through the use of non-conical splice sites [52]. Multiple miRNA map to the LAT region, however, analysis of human trigeminal ganglia suggests VZV does not encode multiple RNA during latency [53,54]. 

A further interesting feature shared by VLT and LAT is their mapping location. Transcription of both VLT and LAT terminates in the repeat region of their respective unique short DNA segments. Both VLT and LAT transcription start sites are located adjacent to the unique short repeats. LAT transcription begins in the internal repeat of the unique long segment, but since this 9212 base pair region in HSV-1 is only 88 bp in VZV, VLT transcription begins within the unique long segment of the virus DNA. Nonetheless, both virus latent transcripts span the long/short junction of the virus genome. Additionally, both latent transcripts map close to the virus origin of DNA replication. VZV spans one of the two origins of VZV DNA replication, while LAT maps approximately 5 × 10^3^ nucleotides downstream of the HSV-1 origin of DNA replication. 

As indicated above, the VLT primary transcript is antisense to VZV ORFs 61 and 62, while LAT maps antisense to HSV-1 RL2, the HSV-1 homology to VZV ORF61. VLT transcripts downregulate the transcription of VZV ORF61 in transfection assays [23]. While it is tempting to attribute similar antisense regulation of RL2 by HSV-1 LAT, repeated findings suggest this is not the case [55,56]. It is more likely that the mechanism regulating LAT transcription also affects the promoter of RL2 [57]. Since no virus-encoded transcription factor is transcribed during latency, the control of latent virus gene transcription must be under host control.

## 5. Epigenetic Control of Alphaherpesvirus Latency

Epigenetics is defined as the process by which a stably heritable phenotypic change results from changes in the chromosome without alterations in its DNA sequence [58]. However, stable phenotypic changes in alphaherpesviruses that are inherited across subsequent virus generations involve mutations of the virus genome [59,60]. Thus, the definition of epigenetics has been enlarged to describe a host-centered mechanism to control virus transcription, especially during the latent stage of the virus life cycle. 

Nucleosomes compact ~150 bp of DNA about two copies each of histone proteins H2A, H2B, H3 and H4. Histones are evolutionally conserved and encoded by intronless genes that use primordial 3’ stem/loop structures instead of 3’ polyadenylation to stabilize the mRNA [61,62,63]. Histone posttranslational modification (PTM) at the 3’ C-terminal domain forms the basis of the “histone code” that has emerged as a fundamental mechanism regulating transcription [64]. Initially, the code associated specific histone PTM to transcriptional outcomes, either actively transcribed “on” (euchromatin) or transcriptionally silent “off” (heterochromatin); however, it is clear that individual histones can exhibit multiple PTM that function in unison to amplify or cancel signals within and between histones as well as form scaffolds for multiple transcription factor binding [65]. Thus, previous studies associating individual histone PTM with transcription status provide a first approximation of the epigenetic landscape but may not describe the entire nucleosome environment. Nevertheless, promoters for VZV ORF63 and 62 genes that are transcribed in human trigeminal ganglia within 24 h post-mortem contain acetylated lysine at position 9 of histone protein 3 (H3K9ac), whereas promoters for ORFs 36 (thymidine kinase) and ORF14 (glycoproteins C) genes that are not transcribed in these ganglia lack H3K9ac [66]. Similarly, the HSV-1 LAT promoter is enriched for H3K4/K9ac during latency when LAT is transcribed compared to virus lytic genes (ICP27 and DNA polymerase) [67], and H3K4 trimethylation (eukaryotic histone PTM) decreases while H3K9me3 (histone heterochromatic PTM) increases as HSV-1 establishes latency [68].

The complex interplay between histone PTM is seen during early-stage virus reactivation when multiple HSV-1 [69,70,71,72] and VZV [20,22,73,74,75] genes of all kinetic classes are transcribed independent of preexisting virus transcription factors. In response to neuronal stress, host c-Jun N-terminal kinase phosphorylates serine residues are neighboring lysine residues on histone protein 3 that contain trimethylation PTM [76,77]. Thus, stress modifies (H3K9me3 + S10) to (H3K9me3 + S10P) and (H3K27me3 + S28) to (H3K27me23 + S28P). The resulting methyl/phosphor switch lowers repressive polycomb group protein binding efficiency and reduces RNA polymerase II containing phosphorylated serine at the 5th position in the C-terminal domain repeat (paused RNA pol II), which is now free to progress through histones that are otherwise non-permissive for transcription [78]. While it is tempting to suggest this scenario provides the mechanism that initiates stage 1 of HSV-1 reactivation, which occurs in the absence of de novo protein synthesis [70,71], the experiment that shows the presence of paused RNA pol II on latent virus DNA is yet to be conducted. Nonetheless, the fact that virus latency and reactivation is associated with specific modifications of bound histones has opened a new avenue for therapeutic developments [79,80,81,82].

CpG islands are defined segments of DNA where this dinucleotide occurs at a higher than random frequency [83]. Methylation of cytosine within CpG islands located in gene promoters is a host mechanism predominately associated with transcriptional silencing in a tissue-specific manner [84]. Abundant 5-methylcytosine within CpG dinucleotides was detected by methyl-specific restriction endonuclease analysis of HSV-1 DNA extracted from quiescently infected lymphoblastoid T cells [85]; however, no 5-methylcytosine was detected by methyl-specific restriction endonuclease or DNA sequence analyses of bisulfate-treated HSV-1 DNA extracted from latently infected mouse trigeminal ganglia [67,86]. Thus, the consensus is that CpG island methylation, the most basic form of transcriptional control via epigenetics, is not involved in HSV-1 gene silencing during latency in neurons of the peripheral nervous system. Computer-assisted analysis of VZV DNA identified 61 CpG islands, with an average length of 415 nucleotides [87], of which 16 are within predicted VZV gene promoters. Thus, while it is generally assumed VZV gene silencing does not involve methylation of CpG islands, the experimental data are lacking.

**Figure 1:**
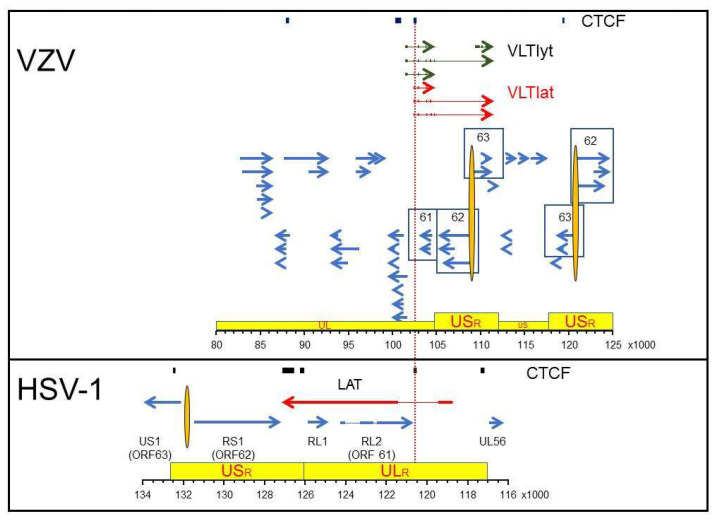
Latency associated transcription regions of VZV and HSV-1. The regions of VZV (top panel) and HSV-1 (bottom panel) that span predicted (VZV) or discovered (HSV-1) CTCF-binding sites are shown relative to the most current VZV transcription annotation (Braspenning et al. 2020) and the annotated HSV-1 reference sequence (NCBI Reference Sequence: NC_001806.2). The genomes are presented with the latency associated transcripts on the top strand in 5’ to 3’ orientation. Arrows show the relative size and direction of each transcript with exons (thick arrows/lines) and introns (thin lines). The red colored latency associated transcripts are distinguished from the blue lytic gene transcripts with the VZV Varicella Latency Transcripts associated with initial stage of virus reactivation colored green (VLTlyt). Specific genes mentioned in the text are identified by number (VZV) or name (HSV-1) with variants of associated genes contained within boxes. The topological architecture of each genome is shown in yellow boxes with the unique long (UL), unique short (US) and associated repeat regions (ULR, USR) highlighted. The nucleotide position of the selected DNA regions is listed for each virus in the ruler at the bottom of each box.

Supplementing and extending studies that describe the nucleosome content of latent virus DNA are the analyses into how these structures are maintained in the context of genomic topology. As a result, it has emerged that control of transcription increasingly focuses on the 3-dimensional structure of gene sets. CCCTC-binding factor (CTCF) is a site-specific DNA binding protein that has been called the “master weaver”, in that it has emerged as the key element maintaining linear domains (enhancer blocker and boundary elements) and intrachromosomal as well as interchromosomal loops [88,89,90]. HSV-1 DNA contains multiple CTCF-binding sites that are occupied during latency, function in a cell-type specific fashion to block the LAT promoter from activating ICP0 transcription and maintain correct demarcation of heterochromatin and euchromatin [91,92,93,94]. The importance of CTCF site occupancy in maintaining virus latency is shown in that the binding of CTCF along with its associated polycomb group protein diminishes upon the induction of reactivation, and also the global reduction of host CTCF transcription induces HSV1 reactivation [95,96,97]. Restricting computer-assisted searches for CTCF-binding sites on the VZV genome to the homologous region on the HSV-1 genome where CTCF occupancy was experimentally determined, identified four potential CTCF-binding sites on the VZV genome [98,99]. Most intriguing is the HSV-1 CTRL2 binding site, which is required for H3K27me3 deposition along with the establishment, maintenance and reactivation of latency [94,95,96,100]. This site is located within the first intron of HSV-1 LAT (Figure 1). A predicted CTCF binging site is present within the first intron of VZV VLT (Figure 1). While evidence suggests this CTCF-binding site in VLT plays a critical role in virus latency and reactivation, its function as a site of CTCF-binding has yet to be reported.

## 6. Immunological Aspects of Alphaherpesvirus Latency with a Focus on VZV

### 6.1. Immune Protection against VZV Infection and Reactivation

The precise immunological determinants of protective immunity against VZV have remained incompletely understood. This is partly because VZV is a strict human pathogen and only limited in vivo data from (humanized) mouse models are available. In this setting, the study of human inborn errors of immunity conferring increased susceptibility to VZV have proven particularly valuable [101,102]. It is clear that a prominent role is played by cellular immunity conferred by T cells and natural killer (NK) cells [103] Regarding the contribution from humoral immunity, the picture is less clear, and VZV infection and reactivation does not seem to be a major problem in individuals with antibody deficiencies. However, a series of previous seminal clinical studies from the VZV pre-vaccine era attest to a significant protective role of VZV immunoglobulins (VZIG) when given to children at risk of severe VZV infection. VZIG was prepared from donors with recent zoster and high levels of VZV specific immunoglobulins, and a small clinical study demonstrated that VZIG prevented infection when given to healthy children shortly after exposure to chickenpox from siblings [104]. Subsequently, clinical trials demonstrated that VZIG administration in immunosuppressed children modified rather than prevented VZV infection, resulting in decreased morbidity of chickenpox in children with leukemia [105,106]. As a consequence of these findings, VZIG was widely used to protect children with cancer after exposure to varicella in the United States and Europe in the period 1975–1995 [107]. Importantly, all types of interferons (IFNs) show antiviral activity against VZV, and serve essential functions in restricting VZV replication and spread, particularly during the viremic phase and in the skin during varicella, and, possibly, also in maintaining latency in sensory neuronal ganglia [103]. While some studies suggest a greater antiviral role of type II IFNs (IFNγ) over type I IFNs (IFNα/β) against VZV in vitro [108], observations from patients with VZV CNS infection, pneumonitis or disseminated skin eruption/zoster in the context of defective Type I or Type II IFN pathways provide more indirect evidence of important non-redundant roles of both Type I and type II IFNs in protecting against VZV reactivation in humans [101,102,109,110]. However, despite several studies addressing these issues, the precise immune cells and immune mediators required for protective immunity in primary infection versus reactivation have not been clarified. In particular, the individual contribution from different cell types, including lymphocytes, macrophages, plasmacytoid dendritic cells and epithelial and endothelial cells, which are all present in human ganglia, remains insufficiently understood and explored.

### 6.2. Immune Histology in Ganglia

Some information has been gained from studying the ganglionic histopathology from 15 individuals with latent HSV-1 or VZV post-mortem and comparing trigeminal ganglia (TG) with dorsal root ganglia (DRG) [111]. When evaluating viral latency transcripts (LAT for herpes simplex virus-1 (HSV 1) and ORF62 from VZV), the authors found evidence of LAT expression in TG but not DRG for HSV-1, whereas ORF62 was detected in both TG and DRG in the case of VZV. A prominent T cell infiltrate was present in TG as opposed to DRGs and the chemokine RANTES was expressed exclusively in TGs (and not in DRGs) for both alphaherpesviruses, indicating antigen recognition and T cell stimulation in TGs. This study was from 2006, and the evaluation of VZV ORF63 might have provided a more precise picture of VZV reactivation. A more recent study examined paraffin-embedded sections from ganglia obtained post-mortem from a patient with PHN obtained years after the herpes zoster rash [112]. Detailed immunological evaluation revealed the presence of VZV DNA as well as an immunological cell infiltrate composed of CD4 T cells, CD8 T cells and CD20 B cells, thus providing somewhat surprising evidence of an ongoing immunological reaction and inflammation years after the reactivation of VZV from latency. These and other studies lend support to the notion that some degree of chronic low-grade reactivation of alphaherpesviruses from latency, as well as immune reactions to this event, does take place over time in ganglia latently infected with VZV

### 6.3. Inborn Errors of Immunity with Genetic Defects Conferring Increased Susceptibility to VZV Infection and Reactivation

Seminal studies on the genetics of inborn errors of immunity (IEIs) or primary immunodeficiencies (PIDs) in humans conducted over the past twenty-five years have revealed monogenic defects in the Toll-like receptor (TLR)3 pathway associated with susceptibility to herpes simplex encephalitis in children and adults [101]. Following these discoveries, additional IEIs have been shown to predispose to severe disseminated primary varicella, frequent and extensive zoster or varicella pneumonia [101,102]. The most prominent example is severe combined immunodeficiency (SCID), in which extensive disseminated viral infections are well-recognized. Severe infection with herpesviruses, including VZV, HSV and cytomegalovirus (CMV) was originally described in a patient with NK cell deficiency as part of a condition demonstrated several decades later to be caused by genetic variants in the myeloid transcription factor GATA2 and named MonoMAC [113,114]. Disseminated and/or recurrent VZV infection can also be a clinical presentation in various other PIDs involving the reduced number or function of one or more subsets of T cells and/or NK cells, in some cases affecting also B cells/all white blood cell subsets, including defects in DOCK2, DOCK8, MHC II, CARMIL2, ORA1/STIM1, STK4, CXCR4, CARD11, CTPS1, MCM4, GINS1, RTL1, FCRF3AMAGT1, CD27, CD70, STAT5B and POLD1 [101,102]. Finally, defects in macrophage intracellular clearance of viruses and mycobacteria, such as those affecting IFNγ receptor, TYK1 and STAT1, may predispose to severe VZV infection/reactivation [101,102].

In 2017, the identification of an autosomal dominant loss-of-function pol III defect in four otherwise healthy children with severe VZV encephalitis or pneumonitis as part of primary varicella was reported [115]. RNA polymerase III (pol III) is a 17 multi-subunit enzyme with a house-keeping function in transcribing rRNAs and tRNAs in the nucleus, but also, since 2009, recognized as serving as an innate immune sensor of AT-rich DNA in the cytosol [116]. The enzymatic activity of pol III has the ability to convert AT-rich DNA into 5’triphosphorylated RNA, which serves as a ligand for another cytosolic pattern recognition receptor, RIG-I, and leads to the production of IFN [116,117]. The inherited defects in pol III in these children were localized in the genes encoding *POLR3A* and *POLR3C* subunits, and it was demonstrated that these missense mutations caused an inability to convert the AT-rich DNA as present in the VZV genome into 5’triphosphorylated RNA; this led to defective type I and III IFN responses to DNA and VZV infection as well as increased viral ORF expression in patients’ PBMCs. Moreover, reconstitution of patient cells with WT POLR3A and/or POLR3C allowed DNA sensing, type I IFN production and viral control comparable to healthy control cells, thus functionally validating the disease-causing potential of the gene variants [115]. This work suggests an important contribution of innate immunity to antiviral defenses against VZV through recognition of the AT-rich VZV genome by pol III and demonstrates a major role of type I IFN I primary VZV infection. Further insight with relevance to VZV reactivation was gained by a subsequent report describing a variant in another pol III subunit, *POLR3F*, in monozygotic adult twins with recurrent VZV meningoencephalitis [118]. In young adulthood these female twins both experienced repeated episodes of CNS vasculitis presenting in a stroke-like manner with hemiparesis, sensory deficits and headache, and clinically diagnosed as recurrent VZV meningoencephalitis with vasculitis. Subsequent studies from our group investigating genetics and VZV-induced immune responses in adults with VZV encephalitis caused by VZV reactivation also identified defects in pol III [119,120]. These publications, together with the twin study on VZV CNS vasculitis, suggest that pol III is important in protection against VZV, not only during primary VZV infection but also potentially in controlling latency during viral reactivation, although the molecular details and full pathogenesis remain to be demonstrated in vivo. 

Among several unresolved questions related to VZV pathogenesis and immunity to this virus is the major issue as to whether pol III plays a direct role in controlling VZV latency through the production of type I and III IFNs, or whether pol III may be involved in the maturation of adaptive immunity during primary infection in more indirect/subtle ways. In the former case, it is not clear whether the role of pol III is exerted in the peripheral sensory ganglia, i.e., the PNS, or rather after reactivation into the CNS. Although several pertinent questions remain, these and other studies provide evidence that type I (and III) IFNs play important roles in VZV infection and reactivation. Intriguingly, in a clinical observation of a patient with disseminated VZV eruption/varicella who had circulating autoantibodies against IFNα, it was first reported in 1981 [119] that type I IFN has an essential role in human protection against VZV reactivation and in neutralizing antibodies against IFNα. Furthermore, IFNγ has, since then, been found in several patients with VZV [102,109,110].

## 7. Role of Autophagy in Reactivation of Other Alphaherpesviruses, the Example of Mollaret’s Recurrent Lymphocytic Meningitis

The majority of VZV disease manifestations associated with PIDs involve increased susceptibility to both primary disseminated infection and reactivation, in the latter case to cause herpes zoster or CNS involvement in the form of meningitis, encephalitis and vasculitis. It may, therefore, be informative to consider a medical condition involving exclusively alphaherpesviruses reactivation. Mollaret’s meningitis, the molecular and immunological pathogenesis of which is largely unexplored, is such a condition, and consists of recurrent lymphocytic meningitis caused by HSV-2 reactivation, in rare cases by VZV or HSV-1 [121,122,123]. Whole exome sequencing of patients with Mollaret’s meningitis recently led to the identification of rare mono-allelic missense variants in the autophagy genes *ATG4A* and *LC3B2* in two adult patients and was the first study to associate defective autophagy with viral infection in humans [124,125]. HSV-2 infection of fibroblasts from these patients demonstrated impaired virus-induced autophagy as well as increased viral replication and enhanced cell death, and this cellular phenotype was reconstituted/rescued to normal by expression of WT ATG4 and LC3B2 in the two patients, respectively [125]. These findings revealed a role for autophagy in antiviral defense and suggest that defective autophagy represents a rare inborn error of immunity associated with susceptibility to HSV2 CNS infection in humans. 

Based on these findings, it is tempting to speculate that autophagy may also play a role in maintaining VZV latency, and that autophagy defects in humans may predispose to severe VZV infection and reactivation from latency. Indeed, in vitro studies have demonstrated both pro-and antiviral roles of autophagy in VZV immunity and pathogenesis. Briefly, several studies have provided evidence of interactions between VZV replication and autophagy pathways during lytic infection and shown that these virus–host interactions take place in a highly cell-type dependent manner. Whereas autophagy seems to exert antiviral roles in some contexts, the virus may also directly utilize autophagy molecules for its own benefit to enhance replication and viral egress (related to the endoplasmic reticulum and other cell organelles) and, thus, evade autophagy and subvert this process [126,127,128,129]. The finding that VZV directly inhibits autophagosome–lysosome fusion may suggest that autophagy does indeed play an important antiviral role in VZV infection, thus explaining/justifying the evolutionary adaptation of the virus to subvert autophagy processes [128]. However, specific knowledge concerning a possible role of autophagy in maintaining VZV latency in sensory ganglia in humans is lacking. Collectively, further studies are needed to resolve the specific role of autophagy in HSV and VZV infection and to define to what extent altered cellular homeostasis and autophagy processes may influence VZV reactivation and disease. 

## 8. Conclusions

During the last decade there has been a significant increase in our detailed knowledge of the complex mechanism underlying VZV latency, as well as an emerging appreciation of the diversity of the clinical syndromes caused by VZV reactivation. The advances in the understanding of VZV latency have been extensive but largely incremental, and a true paradigm shift has yet to be defined. This increased understanding has largely been driven both by new molecular virological technologies and an increased interest in viral latency among the scientific community. The emergence of epigenetic mechanisms influencing VZV latency is likely to lead to an explosion of interest in this aspect. There has also been much recent interest in immunological aspects of VZV latency, mainly in terms of determining the host immune response to the virus and the role of immune protective mechanisms in preventing viral reactivation. This knowledge has been gained, in particular, by studying the clinical manifestations together with the genetics and cellular immune phenotype of individuals with inborn errors of immunity presenting with severe disseminated VZV infection. Since alpha human herpes virus latency is established so early in life, it is unlikely that viral latency can be completely prevented, but a significant consequence of current research is more likely to lead to therapies that may treat or prevent viral reactivation in both immunocompetent and immunocompromised individuals.

## Data Availability

Not applicable.
